# Piloting the Extension for Community Healthcare Outcomes (ECHO) Pediatric Oncology Telehealth Education Program in Western Kenya: Implementation Study

**DOI:** 10.2196/59776

**Published:** 2025-06-03

**Authors:** Tyler Severance, Gilbert Olbara, Festus Njuguna, Martha Kipng'etich, Sandra Lang'at, Maureen Kugo, Jesse Lemmen, Marjorie Treff, Patrick Loehrer, Terry Vik

**Affiliations:** 1 Department of Pediatrics, University of Missouri, 400 N Keene St, Suit 111, Columbia, MO, 65201, United States, 1 573-882-3961; 2 Department of Pediatrics, Indiana University, Indianapolis, IN, United States; 3 Moi Teaching and Referral Hospital, Eldoret, Kenya; 4 Moi University, Eldoret, Kenya; 5 Academic Model Providing Access to Healthcare, Eldoret, Kenya; 6 Department of Pediatric Oncology, Emma Children’s Hospital, Amsterdam UMC, Vrije Universiteit Amsterdam, Amsterdam, The Netherlands; 7 Princess Máxima Center for Pediatric Oncology, Utrecht, The Netherlands; 8 School of Education, Indiana University, Bloomington, IN, United States; 9 School of Medicine, Indiana University, Indianapolis, IN, United States

**Keywords:** pediatric cancer, global health, education, Extension for Community Healthcare Outcomes, project ECHO, pediatric oncology extension, pediatric, oncology, health care, outcome, telehealth, children, cancer, diagnosis, epidemiology, telementoring, technical assistance, virtual platform, effectiveness, equitable access, early diagnosis, collaboration, training, medical education, remote education, online education, low-middle income country, LMIC, community health care outcomes

## Abstract

**Background:**

Childhood cancer has an annual incidence of 150‐160 cases per million children worldwide but remains vastly underdiagnosed in low- to middle-income countries such as those in Sub-Saharan Africa. The Moi Teaching and Referral Hospital (MTRH) serves a population of 25 million people, including 10 million children. The average number of pediatric cancer diagnoses was 216 cases annually in 2017‐2019, which was well below the anticipated 1500 cases based on epidemiology data. The remaining 75%‐80% of pediatric cancer cases remain undiagnosed, and these patients are not likely to survive. Prior outreach and needs assessments demonstrated a lack of medical knowledge related to pediatric cancer as a primary barrier to improved referrals, diagnoses, and ultimately, cure.

**Objective:**

This study aimed to address disparities in medical knowledge contributing to low diagnostic rates of cancer in children. We implemented Project ECHO (Extension for Community Healthcare Outcomes)—a validated virtual guided practice and telementoring model—to connect multidisciplinary specialists at MTRH with staff in medically underserved communities in western Kenya for training, technical assistance, and mentorship.

**Methods:**

Sessions were freely available on Zoom twice monthly and featured an expert-led didactic topic followed by a learner-led, case-based discussion. The discussion used dialogue education to promote learning and engagement among participants, with mentorship from the expert team. Information on ECHO participation was tracked, and electronic surveys were sent to the participants at the end of the pilot year. The ECHO program was run in parallel with the pediatric oncology cancer registry to monitor trends in diagnostic rates within the referral region.

**Results:**

The ECHO program launched successfully in January 2020 with a curriculum focused on pediatric oncology for health care providers. A total of 22 sessions were conducted, with an average of 23 learners per session. A total of 148 participants attended at least one session, with the majority (n=80, 54.1%) attending multiple sessions. The year-end analysis in January 2021 demonstrated that 286 new pediatric patients were diagnosed with cancer at MTRH, representing a 33% increase over the 3-year average.

**Conclusions:**

The Project ECHO platform created a dynamic virtual platform to continue to engage stakeholders across western Kenya. The implementation of this telehealth education platform in Kenya represents an effective model for increasing the recognition and earlier referral of childhood cancer in low- to middle-income countries.

## Introduction

### Pediatric Oncology in Kenya

**Figure 1.**
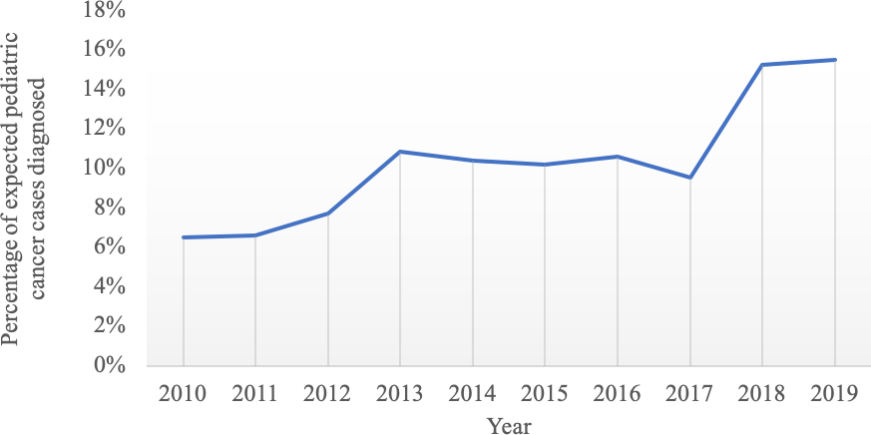
Number of cases of pediatric cancer diagnosed at Moi Teaching and Referral Hospital as a percentage of the expected total number of cases based on cancer epidemiology for the referral region. The pediatric oncology program within Academic Model Providing Access To Healthcare began in 2010, which is when diagnostic data was first available.

Over the past decade, efforts have been made to identify health care disparities affecting children in Sub-Saharan Africa [1]. It is recognized that pediatric cancer rates are far below what would be anticipated based on treatment records at large referral centers [2]. The western regions of Kenya are home to approximately 24 million citizens, which corresponds to 10 million children under the age of 15 [3]. Based on epidemiological data from similar patient populations in the United States, approximately 1000‐1200 patients were diagnosed with pediatric cancer each year [4]. As the only national multidisciplinary referral center in this region, Moi Teaching and Referral Hospital (MTRH) is uniquely positioned to provide multidisciplinary care for these patients, including chemotherapy, oncologic surgery, and radiotherapy. However, in the past 3 years, an average of only 216 pediatric patients were diagnosed with cancer annually [5]. This suggests that the remaining 75%‐80% of patients remain undiagnosed and likely do not survive (Figure 1). One example is pediatric leukemia, the most common type of pediatric cancer in this region. Although this malignancy should represent 300‐480 cases (ie, 30%‐40% of all pediatric cancer diagnosis), only 40 cases per year have been seen over the last several years [5].

### Kenyan Health care and Academic Model Providing Access To Health care (AMPATH)

To better address the disparities impacting western Kenya, an international partnership has grown to educate and empower Kenyan clinicians. This partnership began more than 30 years ago, initially included Indiana University in collaboration with Moi University College of Health Sciences and MTRH, with an emphasis on treating HIV [6,7]. Since then, AMPATH (Academic Model Providing Access To Health care) has expanded to include multiple North American institutions and a broader focus of acute and chronic diseases, including both pediatric and adult oncology [7]. Pediatric oncology training was further bolstered through partnerships with Princess Máxima Center in the Netherlands and Dutch pediatric oncology colleagues. The educational initiatives initially focused on educating clinicians at MTRH, but have since broadened to include specialty training, fellowships, regional outreach programs, and disease-specific conferences for providers in medically isolated communities [5]. The pediatric oncology program within AMPATH includes a comprehensive fellowship program, more than a decade of outreach training, and international collaboration in the United States and the Netherlands [5,8]. These efforts to improve childhood cancer care were critical in highlighting the significant gap between the actual and anticipated diagnostic rates of pediatric cancer based on population and epidemiologic information available in the referral region. Further, engaging with health care providers (HCPs) and medical leaders at the local county level offered insights into diagnostic challenges such as lack of awareness of signs and symptoms of cancer, and knowing when to refer patients for further evaluation [5].

### Project ECHO (Extension for Community Healthcare Outcomes)

Project ECHO (Extension for Community Healthcare Outcomes) was developed in 2003 by Dr. S. Arora, a hepatologist at the University of New Mexico in Albuquerque [9]. He observed that patients with hepatitis C were arriving too late for treatment due to long wait times, especially in the rural, underserved, and socially disadvantaged population in New Mexico. Additionally, the isolated and overworked primary care practitioners required a new health care delivery model to remedy this problem. In response, Arora et al [9] developed a virtual hepatitis C outreach clinic using videoconferencing and didactic lectures by specialists during weekly interprofessional rounds. In this setting, remote practitioners could present their hepatitis C patients, receive guidance from the specialist team at the University of New Mexico, and subsequently treat patients themselves. Their outcomes showed an identical level of care in the rural and university clinics and led to the development of Project ECHO [9].

The ECHO programs use a telementoring approach for the education of clinicians in a hub-and-spoke model. Providers at spoke sites present cases, but no patients are ever on the network. TeleECHO clinics typically convene twice monthly for 60‐75 minutes, during which case presentations, demonstrations, and didactics are provided and recorded for both synchronous and asynchronous observation. All knowledge is shared in a “learning loop” at no cost to participants [10,11]. Continuing medical education and nursing credits are provided, and education certificates are given based on clinical specialty. The goal of each ECHO varies based on the needs of the spoke sites and the disease type. For cancer related care in a high-income country, similar cancer ECHO programs have focused on prevention, screening, and management of survivorship care [12]. However, little data exists to support the use of the ECHO program in Sub-Saharan Africa for cancer care. This paper outlines the pilot implementation of the Project ECHO model to enhance pediatric cancer care in western Kenya.

## Methods

### Framework of Implementation

The first step in building the pediatric oncology ECHO program was to recruit an expert hub team [13]. This team incorporated personnel from both Kenya and the United States, including American pediatric oncologists and pediatric hematology-oncology fellows from Kenya. Additional Kenyan personnel included pediatric oncology nurse navigators, as well as a dedicated coordinator and facilitator for the program. Key ECHO hub team personnel, including both Kenyan and North American program leaders, participated in virtual or in-person immersion training, which is recommended for all new programs [11,13].

The implementation of the ECHO program was consistent with the traditional model [9,14]. Each session comprised a 20‐ to 25-minute didactic lecture following a curriculum based on the previously expressed needs of rural providers. A case-based discussion typically followed the traditional didactic. This required advanced preparation, as spokes were asked to submit a case for discussion at least 24 hours prior to the ECHO session. The information was summarized into a single page document consisting of pertinent information related to the patient’s presentation. All personalized or identifying information was intentionally removed from all presentation materials. During the session, the spoke presented the case and received follow-up clarifying questions from both the spokes and then the hub team. Subsequently, the spokes and hub participants then offered suggestions on how best to address the proposed clinical questions. Occasionally, when no cases were submitted, the hub team identified actively admitted patients from MTRH inpatient wards for discussion.

### Recruitment and Participation

Prior to launch, spoke sites were recruited based on previously established connections in the region. Outreach attempts were made to county referral hospitals and faith-based hospitals in western Kenya. Any HCP who had recently participated in the annual AMPATH Pediatric Oncology Conference, had participated in localized training efforts, or had recently graduated from medical training programs at MTRH was invited to join the ECHO sessions. Primary recruitment efforts mainly consisted of email and phone calls. Participants were also invited to join a mobile phone messaging service (ie, WhatsApp) to allow for continued engagement and recruitment. These relationships established a core of 10‐12 clinics to participate the initial pediatric oncology ECHO sessions.

Attendance at each ECHO session was captured automatically by the parallel iECHO software. This software, which is available at no cost to all ECHO programs, tracked participation at each session and other variables, including recurrent participation and the total number of unique attendees. This information was readily available both during the pilot year and at the conclusion of the curriculum.

### Evaluation

At the end of the year, a survey was sent to all participants recorded in the iECHO database. The survey used a modified Likert scale to assess multiple variables related to the program including enjoyment, comfort presenting a case, continued participation, ability to diagnose pediatric cancer, recognition of signs and symptoms of cancer, and knowledge growth. Initial survey requests were sent via electronic communication with additional reminders sent via email, mobile app, and phone calls. The results of this survey were accumulated in the Research Electronic Data Capture platform.

In the decade prior to ECHO launch, a formalized process was used to track diagnosis and referral patterns of pediatric patients with cancer. This process was continued throughout the duration of the ECHO curriculum. At the conclusion of the pilot year, the total number of new diagnoses was obtained.

### Ethical Considerations

For patients requiring diagnosis and treatment, informed consent was obtained for each case, and institutional review board approval was granted for the entire duration of the project including the multiyear cancer registry (Moi Teaching and Referral Hospital/Moi University College of Health Sciences Institutional Research and Ethics Committee approval number FAN: 0004025). Written consent from parents or guardians and assent from patients was obtained at the time of initial diagnosis in person by a member of the study team. An interpreter was used when the native language of the patient or guardian was different from that of the consenting team member. All patient information for the registry was deidentified at enrollment. Participants and their families were not financially compensated for enrollment.

For the Project ECHO telehealth sessions, this was considered an educational program, and institutional review board approval was not required for this program, as all surveys were optional and patient information was deidentified. Participants were not paid for their time or survey completion, although continuing education credits were offered to active participants in the ECHO program throughout the year.

## Results

### Framework of Implementation

Following the training of hub team personnel, the ECHO program launched successfully in January 2020 with a curriculum focused on pediatric oncology for general health care workers. The program was conducted continuously during the pilot year with omitted sessions only during national holidays. Feedback sessions were held at 8-week intervals throughout the year to continue to adapt to learner needs.

A curriculum was designed based on interpretations of the prior needs assessment [13]. This included an emphasis on the typical presentations of common pediatric malignancies in the region (Table 1). Of note, general review sessions were incorporated into the didactic schedule, which consisted of high-yield information from preceding sessions followed by an opportunity for spokes to provide feedback.

**Table 1.** Final didactic curriculum for the pilot year of the ECHO^a^ program. The didactic schedule was designed by the hub team, with two sessions left open for topics chosen by the participants.

Session	Didactic title
1	Anatomy of a successful ECHO: yearlong goals for this program
2	Pediatric leukemia: basics of disease
3	Pediatric leukemia: history, exam, and lab findings consistent with leukemia (Part 1)
4	Pediatric leukemia: recent updates in epidemiology and outcomes
5	Pediatric lymphoma: basics of disease
6	Pediatric leukemia: next steps in evaluating (bone marrow testing, flow cytometry, tissue biopsy)
7	Transfusions in children: anemia or something else
8	Session open: didactic topic at request of spoke sites^b^
9	Pediatric lymphoma: differential diagnosis and next steps in evaluating
10	Solid tumors: bone tumors–basics and next steps
11	Pediatric leukemia: physical exam findings consistent with leukemia
12	Oncology emergencies at diagnosis
13	Brain tumors in pediatrics
14	Pediatric leukemia: lab findings consistent with leukemia
15	Abdominal masses: differential diagnosis and next steps
16	Session open: didactic topic at request of spoke sites^c^
17	Pediatric leukemia: history findings consistent with leukemia (Part 2)
18	Pediatric leukemia: standard treatment, timing, recent outcomes
19	Kidney tumors in pediatrics: basics and next steps
20	Difficulties enrolling with national health insurance fund
21	Special patient populations: adolescents and young adults
22	Retinoblastoma: what to know
23	Pediatric leukemia: maintenance therapy and survivorship basics

a ECHO: Extension for Community Healthcare Outcomes.

b Topic selected: splenomegaly–physiology and evaluation.

c Topic selected: Wilms tumor–diagnosis and management.

The curriculum implemented throughout the pilot year closely mirrored the proposed outline at the beginning of the year. Participants ultimately requested that lectures on the physiology and evaluation of splenomegaly and on the diagnosis and management of Wilms tumor be added to the curriculum for sessions 8 and 16, respectively. These topics were common themes relevant in preceding case discussions.

### Recruitment and Participation

**Figure 2.**
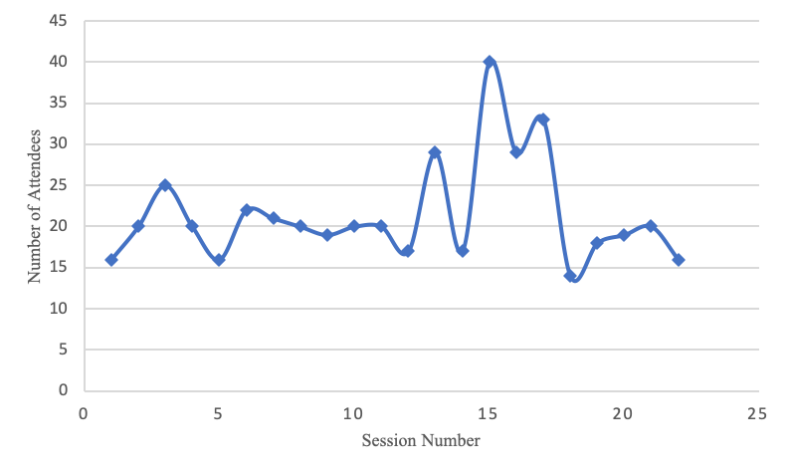
The total participants at each ECHO session during the pilot year are shown. Participation was captured electronically for all individuals logging into the Zoom platform at any time during each of the 22 sessions.

A total of 22 sessions were conducted during the pilot year, with an average of 23 learners per session (Figure 2). This program consisted primarily of staff at hospitals within the referral region. There were 148 total participants who attended at least one session. Among the participants, 80 (54.1%) learners returned and attended more than one session and 38 (25.7%) attended at least four sessions. In addition, during the year, a total of 31 (20.9%) patient cases were presented for discussion, with an average of 1.4 cases per session.

### Evaluation

**Figure 3.**
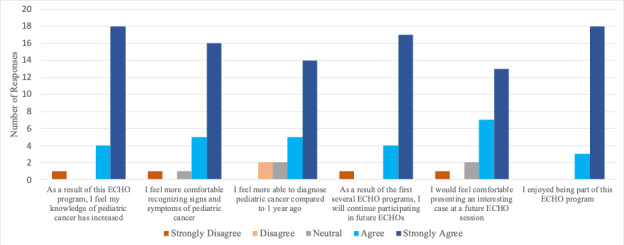
Participant survey results from the pediatric oncology ECHO program following the initial pilot year are shown. Surveys were administered electronically to all participants in the program after the final session and answers were anonymously recorded.

At the conclusion of the pilot year, all survey data was combined and analyzed. A total of 23 (15.5%) participants completed the electronic survey among the 151 total participants. Overall, the majority of responders reflected a favorable impression of the ECHO program (Figure 3).

Finally, the pediatric cancer registry continued to track diagnostic rates during the course of the ECHO pilot. In the calendar year of the pediatric cancer ECHO, a total of 286 new pediatric patients were diagnosed with cancer at MTRH, which represents a 33% increase over the preceding 3-year average.

## Discussion

### Principal Findings

The Project ECHO platform created a dynamic virtual platform to continue engaging stakeholders across western Kenya. The primary goal was to deliver effective education regarding the recognition, diagnosis, and timely referral for pediatric patients with cancer presenting to local health care settings. The combination of strong attendance and favorable participant survey results suggests that the program was effective, particularly within the context of rising pediatric cancer diagnostic rates.

The ECHO program was initially started in New Mexico as a mechanism to improve access to quality care at the population level in high-income countries. While it achieved great success in this capacity, the use of Project ECHO as a mechanism to address disparities in care in LMICs remains a novel prospect. In this setting, the ECHO program was successfully implemented and maintained throughout the pilot year.

The AMPATH collaboration with MTRH, Moi University, and Indiana University, coupled with the expertise offered by the ECHO Institute and the recommended immersion training, was sufficient to launch the program. The prior decade of community outreach and academic conferences provided a strong foundation for the recruitment of spokes from medically isolated communities. In particular, county hospital providers constituted the bulk of participants during the initial pilot year. By using participant-led, case-based discussion alongside a standardized curriculum tailored to the needs of learners, the Project ECHO model led to increased engagement and participation.

As a voluntary educational program, AMPATH Project ECHO placed a large emphasis on measuring recurrent participation as a marker of perceived value from spoke participants. This became particularly valuable in the second half of the year, as attendance continued to rise (Figure 2). This increase also coincided with the start of the COVID-19 pandemic and its eventual spread to rural Kenya. A by-product of the pandemic was a general shift toward virtual platforms in medical communities [15]. With a general increase in comfort with Zoom and telehealth, additional increase in participation was noted. Further, the virtual nature of Project ECHO allowed the program to continue without interruption.

Although the program was available to learners without charge, there were still costs associated with running the platform during the pilot year. The experts on the Hub Team volunteered to participate, and the coordinator of the program received partial funding support through grant sponsorship. Long-term, additional sources of funding will be required to continue running the program effectively or broaden its impact. While the participants could join sessions free of charge, they were not compensated for their time. Many ECHO programs will award continuing education credits or the international equivalent for participation. However, due to funding limitations and logistical challenges, these were not approved for in time to be incorporated during the pilot period.

### Comparison With Prior Work

AMPATH is among the several organizations using the ECHO model to address disparities in cancer care over the past decade [16]. For example, starting in 2018, the ECHO Institute partnered with health care leaders from various African nations to support implementation of the National Cancer Control Plan [17,18]. These efforts included using the ECHO model to support LMICs in navigating the rising cancer incidence. Although there was Kenyan representation at this monthly ECHO, it focused on health care leadership within the Ministry of Health rather than on clinicians at referring hospitals [17].

Other ECHO programs localized within Sub-Saharan Africa, supported by international collaboration have also shown recent success. One such ECHO program focuses on providing education to nursing team members in Cameroon, as they look to improve care for patients with gynecologic cancers and suggests successful implementation based on participation and survey results [19]. Another program focused on the impact of lung cancer and improving recognition of early signs and symptoms by educating doctors and nurses in rural regions of the Northern Cape Province. This group also reported success using the Project ECHO model for training and improving awareness [20].

Expanding beyond acute cancer diagnosis and treatment, additional Project ECHO efforts have focused on supportive care such as the Palliative Care ECHO. A team from the MD Anderson Cancer Center collaborated with colleagues in 5 countries in Sub-Saharan Africa to deliver telehealth education via the ECHO model, with favorable feedback from participants [21]. Another ECHO led by Columbia University and partners throughout Africa focused on improving laboratory practice, collaboration, and resource sharing and has experienced strong participation and growth since its launch in 2018 [22]. Both programs demonstrated successful implementation based on participation and survey results [21,22].

### Limitations

The survey results offer additional insight into the participant experience, although they must be interpreted in the context of the limited response rate. Those participants that completed the survey reflected a general positive experience, as indicated by a majority of respondents selecting “agree” or “strongly agree” for each of the six prompts. This suggests that the knowledge, comfort, and confidence of recognizing and diagnosing pediatric cancer improved during the pilot Project. It is recognized that those with a positive experience may be more likely to complete the voluntary survey associated with the program. However, with voluntary education, enjoyment of the program remains critical to sustained success. The emphasis on creating a pleasant and collegial environment is supported by favorable responses to the final question.

In the preceding years, typical educational interventions focused on annual 1‐ to 2- day conferences targeted at medically underserved communities. Although pre- and post-testing suggest there is potential benefit to be gained from instruction, this knowledge can wane over time–particularly without continued mentorship and active application [23]. The ECHO model was ideal for addressing this gap in instruction as the regularly occurring sessions combined with dialogue-based collaboration and telementorship further augment the growth of local HCPs. However, the generalized registry data reflect trends across the referral region. It is possible that the cumulative effect of other outreach or engagement programs may impact the interpretation of increase in diagnostic rates.

Finally, it is recognized that true assessment of the impact of educational programs such as this one and the impact on public health outcomes is difficult, particularly in a single year of implementation. Future evaluations will aim to correlate involvement and participation in the Project ECHO platform with regional oncology data. For example, trends in diagnosis at the county level, early mortality in cancer care, and even survivorship care could be impacted by sustained education and engagement with local health care teams. Future investigations would also be better positioned to provide comparison or controls by including counties that do not actively participate in the Project ECHO program.

### Conclusions

Given the generalized success of early implementation of the ECHO model, there is significant scope to expand its potential benefits to impact medically isolated communities in Kenya as well as across Sub-Saharan Africa. Further, current evaluations of ECHO programs are largely limited to participation and survey results which are common in educational program assessments. The unique position of MTRH as the primary pediatric oncology treatment center in the region allows for rapid assessment of impact based on direct availability of diagnostic data and allows additional insight into the impact on public health outcomes. During the pilot year, analyses showed an increase in new oncology diagnoses at MTRH and support continued implementation with additional evaluations and analysis in the coming years. Finally, a favorable impact on the pediatric oncology care also suggests the potential to expand ECHO implementation beyond childhood cancer to address other impactful diseases at the regional level.

## Abbreviations

AMPATH: Academic Model Providing Access to Healthcare
ECHO: Extension for Community Healthcare Outcomes
HCP: health care provider
LMIC: low- to middle-income country
MTRH: Moi Teaching and Referral Hospital
